# Evaluation of Machine Learning Methods for Monitoring the Health of Guyed Towers

**DOI:** 10.3390/s22010213

**Published:** 2021-12-29

**Authors:** Diana Marcela Martinez Ricardo, German Efrain Castañeda Jimenez, Janito Vaqueiro Ferreira, Euripedes Guilherme de Oliveira Nobrega, Eduardo Rodrigues de Lima, Larissa M. de Almeida

**Affiliations:** 1 Department of Computational Mechanics, Faculty of Mechanical Engineering, Universidade Estadual de Campinas, Campinas 13083-860, Brazil; germancasta01@fem.unicamp.br (G.E.C.J.); janito@unicamp.br (J.V.F.); egon@fem.unicamp.br (E.G.d.O.N.); 2 Exploratory Hardware Desing Department, Instituto de Pesquisas Eldorado, Campinas 13083-898, Brazil; eduardo.lima@eldorado.org.br; 3 Transmissora Aliança de Energia Elétrica S.A., Rio de Janeiro 20010-010, Brazil; larissa.almeida@taesa.com.br

**Keywords:** guyed tower, finite element, wind loading, machine learning, autoencoder

## Abstract

This paper presents the development of a methodology to detect and evaluate faults in cable-stayed towers, which are part of the infrastructure of Brazil’s interconnected electrical system. The proposed method increases system reliability and minimizes the risk of service failure and tower collapse through the introduction of predictive maintenance methods based on artificial intelligence, which will ultimately benefit the end consumer. The proposed signal processing and interpretation methods are based on a machine learning approach, where the tower vibration is acquired from accelerometers that measure the dynamic response caused by the effects of the environment on the towers through wind and weather conditions. Data-based models were developed to obtain a representation of health degradation, which is primarily based on the finite element model of the tower, subjected to wind excitation. This representation is also based on measurements using a mockup tower with different types of provoked degradation that was subjected to ambient changes in the laboratory. The sensor signals are preprocessed and submitted to an autoencoder neural network to minimize the dimensionality of the resources involved, being analyzed by a classifier, based on a Softmax configuration. The results of the proposed configuration indicate the possibility of early failure detection and evolution evaluation, providing an effective failure detection and monitoring system.

## 1. Introduction

In recent years, the growing demand for electricity and the wealth of water resources in the Brazilian territory have led to an increased adoption of guyed towers for the distribution network due to their lower cost. Along with the growth in demand, a significant number of accidents with this type of structure has occurred. The collapse of guyed transmission towers is one of the causes of service failure in the national interconnected electricity system. The specific periodic inspections carried out in all towers of a concessionaire during the year are not sufficient to detect and predict faults in these towers until the next inspection, and involved complex and expensive operations. Therefore, in this study, we constructed an artificial-intelligence-based monitoring methodology to implement an effective predictive maintenance to avoid the occurrence of faults, and eventual tower collapse, benefiting the end consumer with stable service.

The development of innovative continuous monitoring methods are a current engineering research important topic, based on the state identification of each monitored asset, through online measurements and signal processing, consequently assessing their health state in order to make reliable failure prognostics. The overall expected result is an improvement in maintenance costs, a significant reduction in field inspections and an increase in the reliability of the system and the quality of service.

Given this problem, many studies have been aimed at assessing the structural condition of guyed towes. Initial targets were wind-induced vibration analysis towers coupled to transmission lines [1], and analytical-numerical modeling for structural analysis in non-cable-stayed towers under the action of the wind and stability control of the tower [2]. Other research focused on structural failures related to weather conditions and highlighted the importance of studying the dynamic response of cable-stayed towers for transmission lines under wind loading [3].

Considering the complexity of mathematical modeling the faulty behavior of the towers, machine learning (ML) approaches were applied successfully to the identification of faults in electrical distribution systems [4]. These algorithms were also used to detect structural failures in cable anchored rods in electrical towers [5] and to detect failures due to errors in the anchor installation [6].

This paper proposes a methodology to detect incipient failures and assess their severity in order to avoid the occurrence of a structural collapse. For this dual purpose, data-driven models of normal behavior were created and trained using two autoencoder and classifier neural network configurations, based on simulated and experimental datasets. These models were then used to detect anomalies through comparative analysis of a testing subset of signals from the datasets. These resulting residual signals were used to characterize different cable-stayed tower models. The simulated dataset was from a finite element model, developed for the original tower design, and the experimental data were acquired from a laboratory mockup tower. The ML models were trained considering different wind excitations for normal and faulty states of the cables that stabilize the towers.

The remainder of this article is organized as follows: In Section 2, the fundamentals of the adopted autoencoder and classifier are briefly reviewed. Section 3 presents the two proposed methodologies for detecting and evaluating failures in cable-stayed towers. The results of the proposed methodologies for classification and location of faults in towers are described in Section 4, and the conclusions are presented in Section 5.

## 2. Theoretical Background

### 2.1. Multilayer Perceptron Network

**Figure 1:**
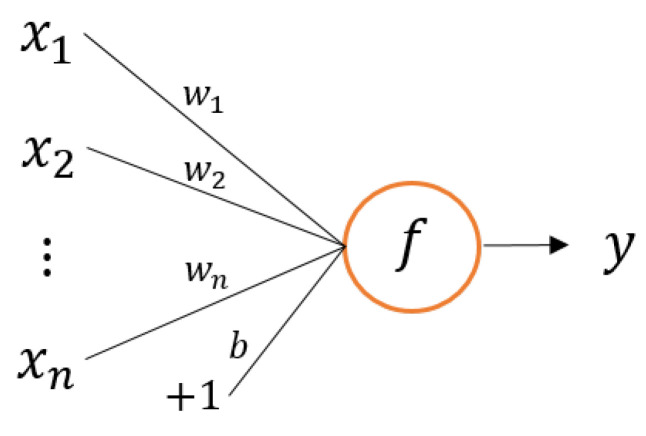
Basic perceptron neuron configuration.

In the field of artificial intelligence, several different artificial neural networks are commonly used. Among them, multilayer perceptron (MLP) was the first network to attain real success in applications, and it is now widely adopted. The simplest MLP configuration is based on three layers: a data input layer, a hidden neuron nonlinear activated layer, and an output layer. The neuron layers are based on the basic perceptron configuration of an artificial neuron, as shown in Figure 1.

The artificial neuron equation is defined as [7]:

(1) $$y\left(x\right)=f\left(\sum_{i=1}^{n}w_{i}x_{i}+b\right)$$

where *n* is the number of inputs, *x* is the set of inputs, *w* is the input weights, *b* is the neuron bias, and *y* is the output of the neuron that is activated by a nonlinear function *f*, for example, the sigmoid function defined by Equation (2):

(2) $$f\left(z\right)=\frac{1}{1+exp\left(-z\right)}.$$

**Figure 2:**
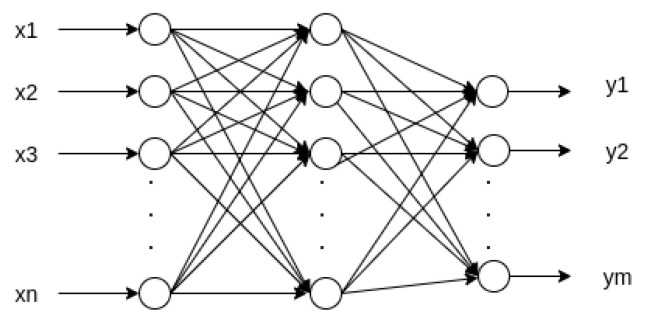
Multilayer perceptron network.

Figure 2 shows the basic three-layer configuration, where a universal approximator represents a nonlinear mapping between an input vector *x* and an output vector *y*. The architecture of a multilayer perceptron with more than three layers is referred to, in general, as a deep MLP.

Multilayer perceptron classifiers have the ability to learn through training in a supervised manner. MLP training generally uses the backpropagation technique, performed by retroactively calculating the mean squared error of the output sample against the training dataset labels, according to Equation (3),

(3) $$J\left(w,b_{int},w,b_{out}\right)=\sum_{k=1}^{N}{\left(d_{k}-\hat{y_{k}}\right)}^{2},$$

where $d_{k}$ a the desired value and $y_{k}$ is the network output for each sample. Training data are presented repeatedly and net weights are adjusted by backpropagation until the desired input–output mapping error falls below a small specified threshold.

### 2.2. Autoencoders

An autoencoder is an unsupervised training artificial neural network that reconstructs the input signal at the output, learning an intermediate model of the input data, in general, with a lower-dimensional representation [8]. They are used in several applications, such as reducing the dimensionality of the data, detecting anomalies, and extracting data characteristics, among others [9,10,11].

An autoencoder consists of two main modules: an encoder and a decoder. The encoder compresses the input into a smaller space, learning the representation of the input data. The decoder reconstructs the input data based on the encoder representations [12].

**Figure 3:**
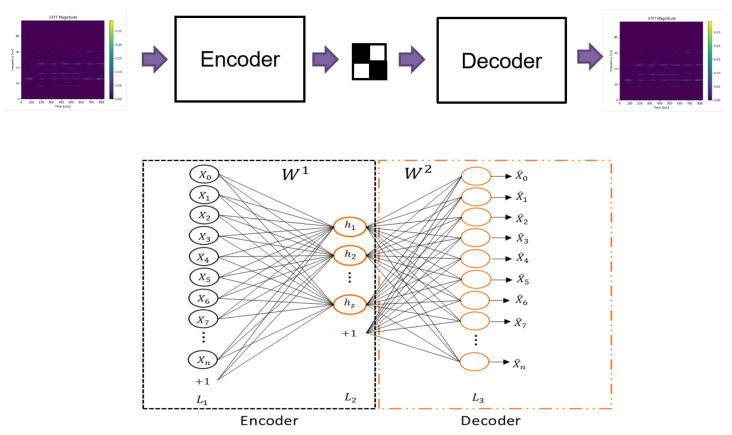
Autoencoder architecture.

It is possible to observe the architecture of an autoencoder in Figure 3, composed by its two modules, with the resulting model in the center. The encoder transforms the *x* input into its representation *h*, and the decoder inversely transforms the representation *h* into the reconstruction output $\hat{x}$. In addition, the architecture of a neural network with unsupervised learning with three layers is shown, where $L_{1}$ is the layer that represents inputs, $L_{2}$ is the hidden layer that represents the learned model, and $L_{3}$ is the output layer with the same dimension as the input layer that represents the reconstruction [13].

Both the encoder and the decoder may be any type of artificial neural network; however, they must have with symmetrical layers in general. For the autoencoder in Figure 3, the encoder output is therefore defined by Equation (4):

(4) $$h=f\left(\sum_{i=1}^{n}W_{i}^{1}x_{i}+b_{1}\right),$$

where *x* represents the *n*-input vector, $W^{1}$ is the weight matrix, $b_{1}$ is the bias, and *f* is the activation function. The encoder output *h*, a reduced representation of the input data, known as the latent model, is then used to reconstruct the data $\hat{x}$, using Equation (5):

(5) $$\hat{x}=f\left(\sum_{i=1}^{s}W_{i}^{2}h_{i}+b_{2}\right),$$

where *s* is the latent model dimension, $W^{2}$ is the weight matrix, $b_{2}$ is the bias, and *f* is the activation function. Finally, the output layer is given by:

(6) $$\hat{x}=f\left(\sum_{i=1}^{s}W_{i}^{2}\;g{\left[\sum_{j=1}^{n}W_{j}^{1}x_{j}+b_{1}\right]}_{i}+b_{2}\right).$$

Then, the mean square error (MSE) of the reconstructed data $\hat{x}$ is calculated using Equation (7),

(7) $$L\left(x,\hat{x}\right)=MSE=\frac{1}{n}\sum_{i=1}^{n}{\left(x_{i}-\hat{x_{i}}\right)}^{2}.$$

#### 2.2.1. Deep Autoencoder

**Figure 4:**
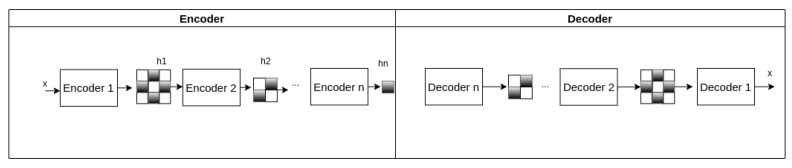
Deep autoencoder architecture.

A deep autoencoder is a multilayer neural network with more than three layers in the encoder and decoder. The deep autoencoder may be trained successively, called a stacked autoencoder, which we adopted in this work. As shown in Figure 4, each stacked encoder’s output vector represents the extracted features that are submitted to the next encoder [14].

#### 2.2.2. One-Dimensional Convolutional Autoencoder

**Figure 5:**
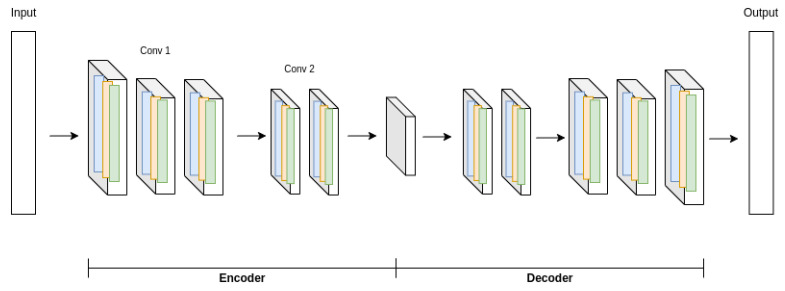
A 1D convolutional autoencoder architecture.

The 1D convolutional autoencoder is a variant of the deep autoencoder, using convolutional layers followed in general by subsampling in the encoder, and transposed convolutional layers and oversampling in the decoder. Convolutional layers are kernel-based, which means that their neurons are not fully connected. They are used to learn hierarchical representations from the input data, which results in particularly effective feature extraction from signals. Figure 5 illustrates a typical 1D convolutional autoencoder architecture [15].

### 2.3. Softmax Classifier

**Figure 6:**
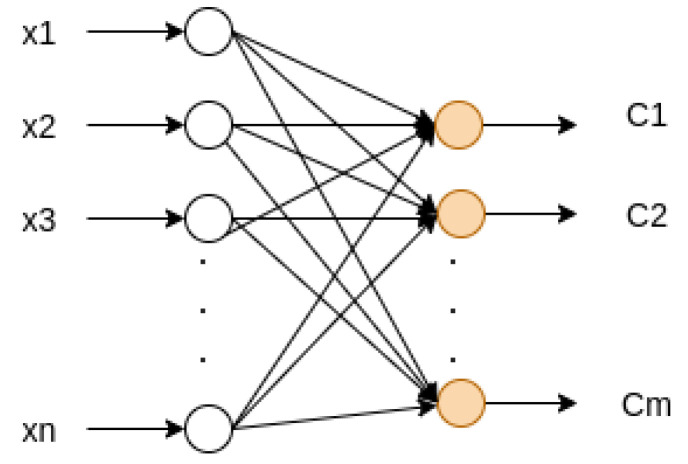
Softmax classifier network.

The Softmax classifier is a fully connected perceptron network used for multiple classes, where the last layer has a logistic regression activation function, with one probability output for each class [16]. Figure 6 shows an example of a Softmax classifier.

Considering Figure 6, for each class output $C_{j}$, where j=1,2,⋯,m, the logistic function represented in Equation (8) corresponds to the probability of each class $C_{j}$,

(8) $$f_{j}\left(y\right)=\frac{e^{y_{j}}}{\sum_{k=1}^{N}e^{y_{k}}},$$

where $y_{j}$, adding to 1, is the weighed sum of $x_{i},\;i=1,2\cdots ,n$.

## 3. Monitoring Configurations

This section describes the data preprocessing and configurations of two different classifying networks, for the finite element (FE) model and the experimental mockup tower, adopted for the case studies. For each case, the behavior of the towers, under normal and faulty conditions, were obtained in response to wind excitations, represented by the measurements of cable-positioned sensors.

**Figure 7:**
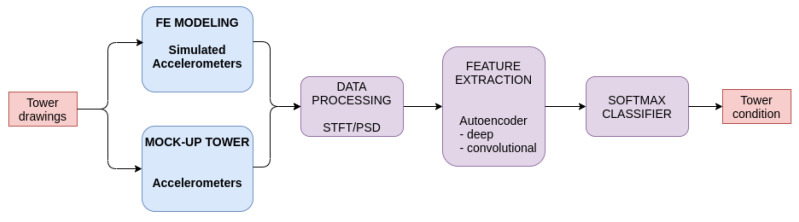
Methodology for tower state analysis.

Figure 7 shows our methodology workflow from model generation to tower condition estimation for implementing a two autoencoder algorithms aiming at constructing architecture based on an autoencoder feature extraction followed by a classifier. The following steps were taken:

- We modeled the tower using the FE method based on the drawings of a real tower;
- Using the FE model, we performed simulated acceleration measurements under normal and faulty operating conditions;
- We conducted experiments and acceleration measurements in the laboratory using the mockup tower under normal and provoked faulty operating conditions;
- The simulated and experimental datasets were divided into two subsets, for training and testing, with a validation subset separated from the training one;
- Acceleration data were preprocessed using short-time Fourier transform (STFT) or power spectral density (PSD);
- Two configurations of unsupervised autoencoder algorithms were implemented and trained for the faulty states;
- We trained a Softmax classifier using the autoencoder latent models as inputs under supervision, with each operating condition as class labels;
- Finally, the test datasets were used to estimate the condition of the tower.

### 3.1. Finite Element Modeling

**Figure 8:**
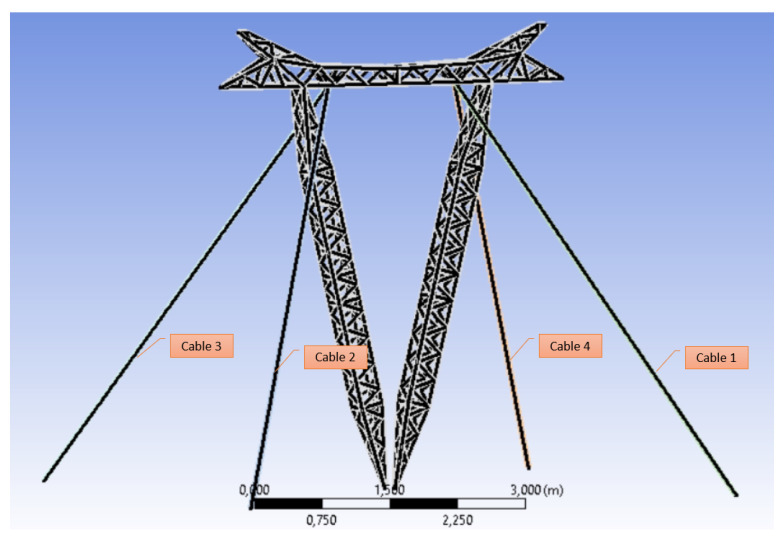
Cable configuration in the finite element model of the tower.

The FE-based model was developed according to the original tower drawings of the cable-stayed towers, provided by TAESA™, with the aim of analyzing the tower’s behavior for different wind speeds and directions, considering the degradation of cable foundations. The basic tower FE model including cable positions is shown in Figure 8.

Simulated time-domain triaxial acceleration signals were obtained in response to wind excitations with a 200 N magnitude. Signals were sampled with a 100 Hz frequency and 120 s of total acquisition time, resulting in 12,000 sample points for each measured node direction. Experiments were performed for five wind directions, considering one normal and two faulty conditions for each separate cable connector foundation.

### 3.2. Experimental Modeling

**Figure 9:**
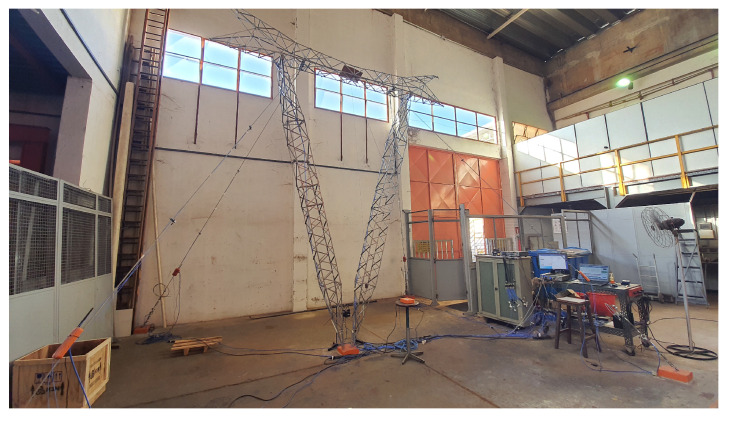
Laboratory mockup tower.

The mockup tower installed in UNICAMP’s LabEDin laboratory is illustrated in Figure 9. Triaxial PCB 356A15 accelerometers were installed on each of the cables to provide vibration signals. A monitoring procedure was defined for the data-acquisition system responsible for measuring and transferring the signals to a database, using pre-established sample frequency, number of samples, and time interval between measurements.

### 3.3. Data Processing

As already mentioned, two different signal processing methods, STFT and PSD, were adopted to generate spectrograms based on simulated and experimental data, enabling time-frequency data analysis.

For the STFT algorithm, the original signals were divided into several parts of equal length using a sliding Hann window, before applying discrete time Fourier transform (DTFT), producing a spectrogram of the signal [17]. The STFT follows Equation (9),

(9) $$X\left(n,\omega \right)=\sum_{m=-\infty }^{\infty }x\left(m\right)w\left(n-m\right)e^{-j\omega m}$$

where x(m) is the discrete time signal, w(m) is the window function, and X(n,ω) is the DTFT of the windowed signal for a short time section. The STFT was calculated using 2000 points in each section segment for a total record of 12,000 samples. An overlap of 50% was used for each section, resulting in 11 spectra for each acquired signal.

### 3.4. Deep Autoencoder Architecture

**Figure 10:**
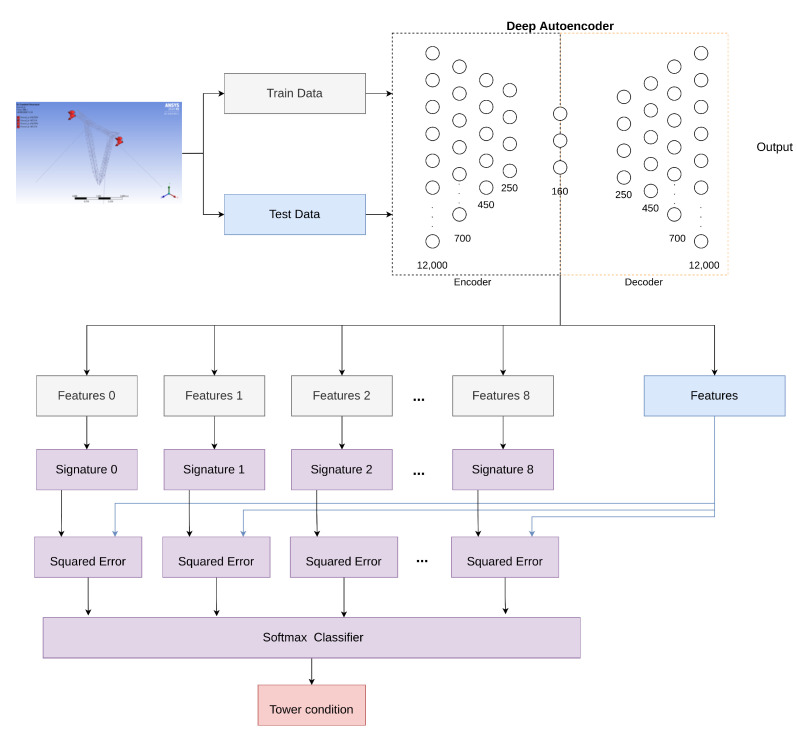
Deep autoencoder methodology.

This subsection details the first proposed configuration for classifying the tower faults for both datasets. The procedure is illustrated through the represented network architecture in Figure 10. Initially, an offline procedure creates the latent models, which begins with signal processing to calculate the spectrograms for the training subsets, and proceeds with the unsupervised training of the deep autoencoder. These latent models are called condition signatures. Then, the Softmax classifier is trained under supervision, having as inputs the mean squared residues obtained through comparison between each signature and the vector of the latent results of the training subset, using the tower conditions as labels. After the training of the complete network, the testing subset is used to generate the mean squared residues and the respective Softmax classification in order to achieve an estimation of the state of the tower for each measurement. The tower state indicates anomaly detection if it is not classified as in a normal state, or a severity diagnosis for the tower behavior.

The deep autoencoder architecture has 12,000 neurons in the first layer, the second layer has 700 neurons, the third layer has 450 neurons, the fourth layer has 250 neurons, and the last layer has 160 neurons, which significantly reduce the dimension of the input data.

### 3.5. Convolutional Autoencoder Architecture

**Figure 11:**
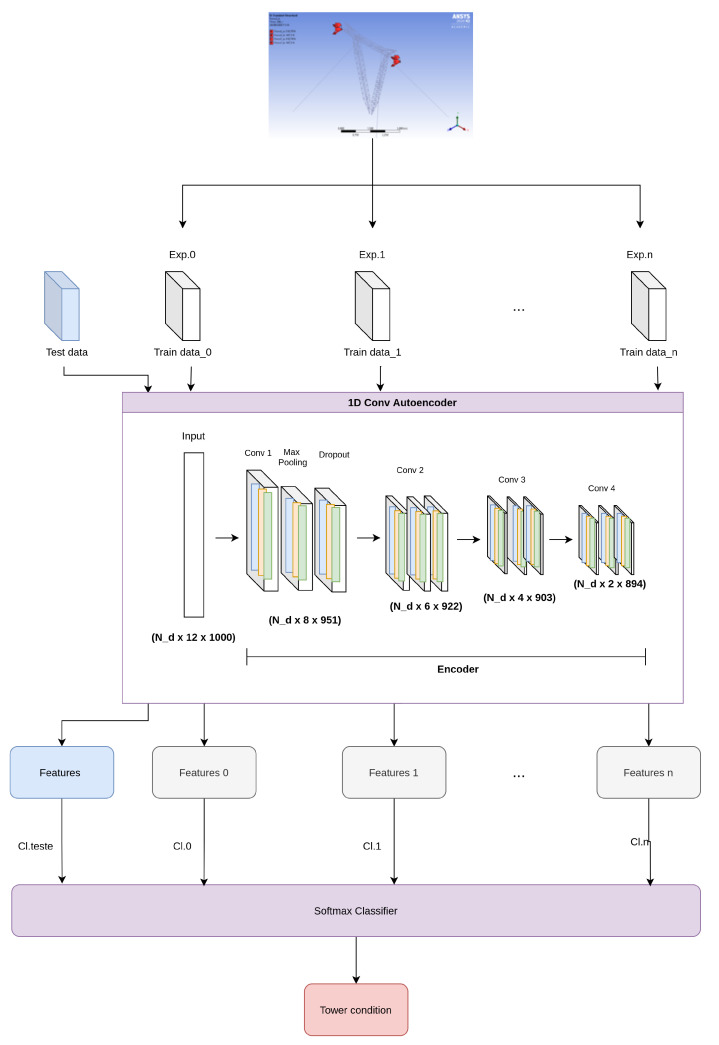
Methodology with 1D convolutional autoencoder.

This subsection details the second method used for classifying tower failures, which is similar to the first one, but adopts a convolutional autoencoder based on 1D kernels instead of a deep multilayer autoencoder. The procedure is illustrated based on the network shown in Figure 11. It starts with processing all the tower vibration data to obtain the spectrograms, which are then separated for training and testing. The first subset is used to train the convolutional autencoder to generate the condition signatures, represented in the figure by the Features blue box. After autoencoder training, the Softmax classifier is then trained using the condition labels. For the test phase, the measured signals are similarly processed by the autoencoder and compared to the signatures to generate the residues, which proceed to the classifier for anomaly detection and severity state diagnosis.

The 1D convolutional autoencoder architecture has a size of 12,000 in the first convolutional layer, a second convolutional layer of 7608, a third convolutional layer of 5532, a fourth convolutional layer with a size of 3612, and a final convolutional layer with a size of 1788. Each convolutional layer has a max pooling and a dropout layer in the encoder.

## 4. Results

This section details the results of the proposed methodologies for locating and classifying tower faults.

### 4.1. Simulated Dataset

Table 1 shows the complete simulated test setup with a total of 45 experiments. Table 1 shows that nine different experiments were carried out, labeled from 0 to 8. Experiment 0 represents the normal tower condition; experiments 1 and 2 represent a fault in cable 1, where it is displaced by 0.002 and 0.004 mm, respectively; experiments 3 and 4 correspond to the same faults, but in cable 2; experiments 5 and 6 correspond to the same faults, but for cable 3; and experiments 7 and 8, for cable 4. The Wind Force column in Table 1 shows the five different wind directions with the same magnitude of 200 N for each cable condition.

### 4.2. Experimental Dataset

**Figure 12:**
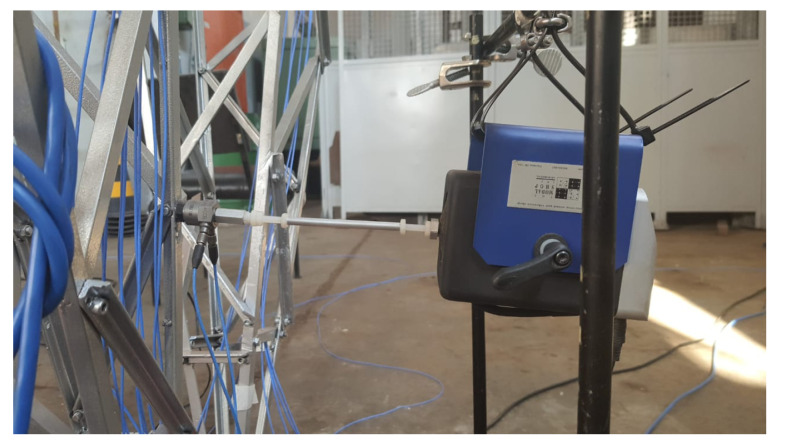
SmartShaker model K2004E01.

To assess the proposed methodology, we used systematic relaxation of the cable tensioners as a procedure to simulate failures in the tower. Table 2 shows that 13 experiments were performed, labeled 0 to 12. Experiment 0 represents the normal tower condition; experiments 1 to 3 represent a failure in cable 1, where cable 1 was displaced in 0.004, 0.008, and 0.012 mm, respectively; experiments 4 to 6 correspond to the same faults, but in cable 2; experiments 7 to 9, for cable 3; and experiments 10 to 12, for cable 4. To construct the tower modes, a shaker manufactured by SmartShaker™, model K2004E01, was used applies a null mean random force with variance of 4.6 N^2^ and a cut-off frequency of 256 Hz, located on the truss near the base, as shown in Figure 12. The tests were carried out periodically for several months in an uncontrolled ambient environment where the temperature conditions changed throughout the day, implying that the measured dataset contains this variation’s influence on the tower cables.

### 4.3. Deep Autoencoder Modeling

**Figure 13:**
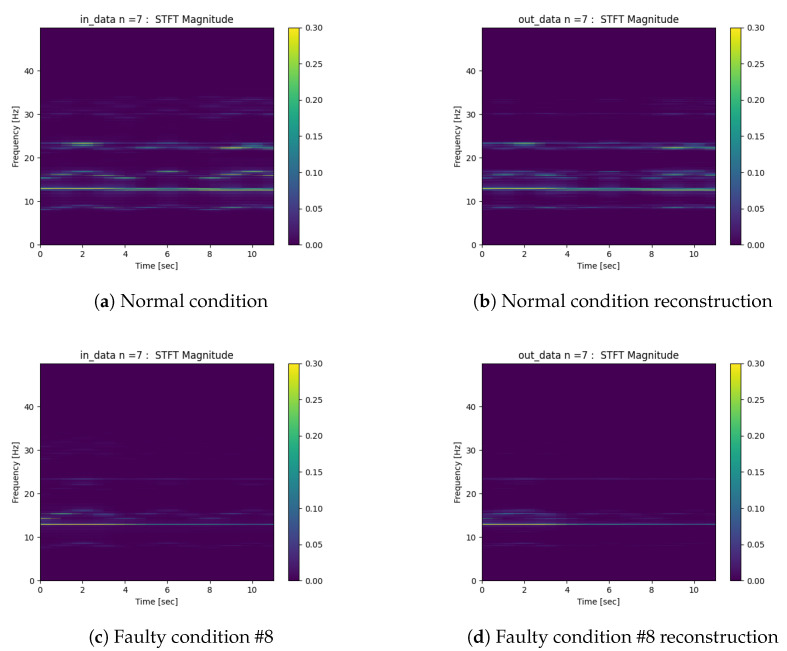
Deep autoencoder examples of reconstructions. (**a**) Normal condition. (**b**) Normal condition reconstruction. (**c**) Faulty condition #8. (**d**) Faulty condition #8 reconstruction.

The deep autoencoder was trained with 60% of the dataset, 20% was used for training validation, and the other 20% was used for testing. The STFT was normalized by its maximum amplitude value. Figure 13 shows the result of the normalized STFT reconstruction of the acceleration signals of the four tower cables, with wind in the A direction, for two conditions (#0 and #8), as detailed in Table 1. The STFT spectrograms in Figure 13 represent the signals in the x,y,z directions, composed in this sequence in the horizontal axis. The left figures show the original signals, and the right figures show the respective reconstruction as produced by the autoencoder. We therefore concluded that the autoencoder models were able to achieve to adequate quality for the reconstruction of the input data. These results were also obtained for the models with other conditions.

The confusion matrix, as shown in Table 3, is a tool commonly used to evaluate classification models. It presents the deep encoder configuration results, where the data in the main diagonal represent the number of successful classification, and the other values correspond to incorrectly sorted data.

The test data in Table 3 show that the classification model was able to completely detect anomalous conditions of the tower, that is, differentiate the normal from the faulty conditions. Additionally, it identified the cable in which the fault occurred and its intensity, with an overall accuracy of 94%. We also verified that all misclassifications occurred for the fault in cable 2 with low severity, which was classified as the fault with high severity.

### 4.4. One-Dimensional Convolutional Autoencoder Modeling

The second proposed architecture was used to analyze the experimental dataset, obtained according to the experiments in Table 2. The data were preprocessed and normalized by the value of maximum amplitude to generate the PSD spectrograms. The models were obtained following the procedure of the 1D convolutional architecture in Figure 11.

**Figure 14:**
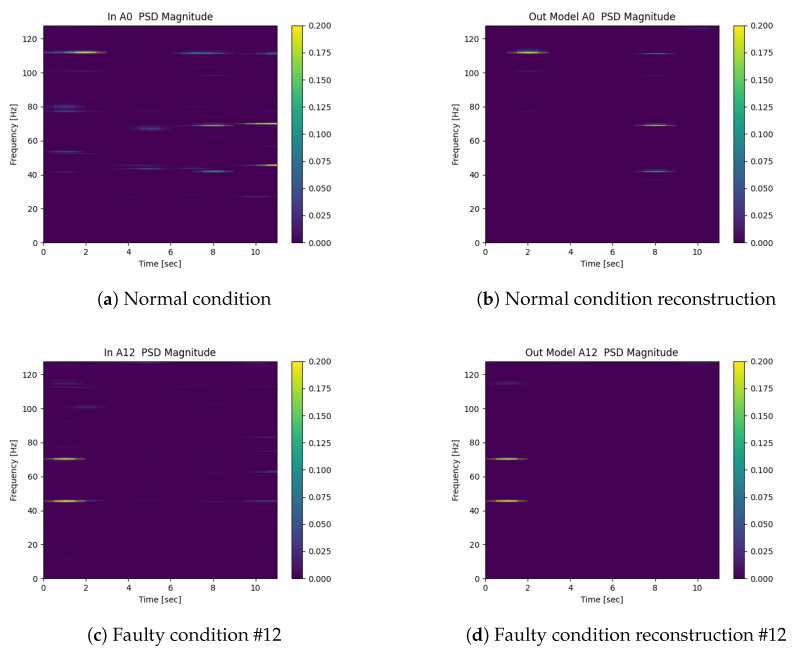
Some 1D convolutional autoencoder examples of reconstructions. (**a**) Normal condition. (**b**) Normal condition reconstruction. (**c**) Faulty condition #12. (**d**) Faulty condition reconstruction #12.

Figure 14 shows the result of the PSD spectrogram reconstruction of the acceleration signals of the four tower cables for two conditions (#0 and #12). The left column shows the normal PSD spectrograms in the x,y,z directions composed in the horizontal axis. The right column shows the reconstructed spectrograms for the two conditions.

Table 4 shows the test data confusion matrix obtained for the 1D convolutional network followed by an MLP classifier with a Softmax output layer applied to the experimental dataset.

Table 4 shows that the classification model completely differentiated the normal condition of the tower from the faulty conditions. It also identified the faulty cable and its severity through classification of its state, with an overall accuracy of 95%. This indicated that this proposed method provides adequate performance with data acquired under real conditions, that is, with measurement noise and varying ambient conditions.

## 5. Conclusions

Here, we proposed a noninvasive approach for anomaly detection and severity estimation for monitoring cable-stayed power transmission towers. The performance of two proposed neural network configurations was analyzed for two datasets, where the first was based on a finite element method of a real tower and the second was based an experimental one with data acquired from a mockup laboratory tower. Vibration excitation was created for sensor measurements under normal and faulty conditions for the two datasets, and machine learning algorithms were developed to assess the tower behavior, aiming anomaly detection and health state estimation.

For both datasets, an architecture based on spectrogram analysis, resulting from processing signals from an accelerometer installed on a cable, was used to train an unsupervised autoencoder, followed by a fully connected multilayer neural network classifier with a Softmax output. For the simulated dataset, an STFT spectrogram with a deep autoencoder configuration was used and, for the experimental dataset, a PSD spectrogram and a 1D convolutional autoencoder both followed by the supervised Softmax classifier.

The proposed methods allows the generation of machine-learning models that identify anomalous conditions of a tower and classify the failure severity, identifying the faulty cable with an overall accuracy of 94% and 95% for the simulated and measured datasets, respectively.

We used the spectrograms obtained with the preprocessing of the tower vibration data to generate a training dataset for the machine learning deep autoencoder algorithm, which achieved an adequate representation of the characteristics of the tower’s behavior under different cable failure conditions. The test results showed the detection of the anomalous vibration behavior of the tower in 100% of the cases, also locating the damaged cable in all cases and estimating the corresponding severity for 94% of the simulated conditions tested and 95% of the experimental tests.

The next steps in this line of work are to extend the application of the architectures and models to real towers in the field. This will be pursued through domain adaptation techniques or a generative adversarial network that allows the generation of new simulated datasets, which are indistinguishable from the experimental signals, and thus can be used to train models that may easily generalize to real towers datasets.

## Figures and Tables

**Table 1:** Tower simulated experiments under normal and faulty conditions.

Experiment	Condition	Cable Displacement (m)				Wind Force (N)			Direction
		1	2	3	4	*x*	*y*	*z*	
		0	0	0	0	0	0	0	–
		0	0	0	0	+177.92	0	+129.40	A
0	Normal	0	0	0	0	+177.92	0	−129.40	B
		0	0	0	0	−177.92	0	−129.40	C
		0	0	0	0	−177.92	0	+129.40	D
		0.002	0	0	0	0	0	0	–
		0.002	0	0	0	+177.92	0	+129.40	A
1	Fault 1	0.002	0	0	0	+177.92	0	−129.40	B
		0.002	0	0	0	−177.92	0	−129.40	C
		0.002	0	0	0	−177.92	0	+129.40	D
		0.004	0	0	0	0	0	0	–
		0.004	0	0	0	+177.92	0	+129.40	A
2	Fault 2	0.004	0	0	0	+177.92	0	−129.40	B
		0.004	0	0	0	−177.92	0	−129.40	C
		0.004	0	0	0	−177.92	0	+129.40	D
		0	0.002	0	0	0	0	0	–
		0	0.002	0	0	+177.92	0	+129.40	A
3	Fault 3	0	0.002	0	0	+177.92	0	−129.40	B
		0	0.002	0	0	−177.92	0	−129.40	C
		0	0.002	0	0	−177.92	0	+129.40	D
		0	0.004	0	0	0	0	0	–
		0	0.004	0	0	+177.92	0	+129.40	A
4	Fault 4	0	0.004	0	0	+177.92	0	−129.40	B
		0	0.004	0	0	−177.92	0	−129.40	C
		0	0.004	0	0	−177.92	0	+129.40	D
		0	0	0.002	0	0	0	0	–
		0	0	0.002	0	+177.92	0	+129.40	A
5	Fault 5	0	0	0.002	0	+177.92	0	−129.40	B
		0	0	0.002	0	−177.92	0	−129.40	C
		0	0	0.002	0	−177.92	0	+129.40	D
		0	0	0.004	0	0	0	0	–
		0	0	0.004	0	+177.92	0	+129.40	A
6	Fault 6	0	0	0.004	0	+177.92	0	−129.40	B
		0	0	0.004	0	−177.92	0	−129.40	C
		0	0	0.004	0	−177.92	0	+129.40	D
		0	0	0	0.002	0	0	0	–
		0	0	0	0.002	+177.92	0	+129.40	A
7	Fault 7	0	0	0	0.002	+177.92	0	−129.40	B
		0	0	0	0.002	−177.92	0	−129.40	C
		0	0	0	0.002	−177.92	0	+129.40	D
		0	0	0	0.004	0	0	0	–
		0	0	0	0.004	+177.92	0	+129.40	A
8	Fault 8	0	0	0	0.004	+177.92	0	−129.40	B
		0	0	0	0.004	−177.92	0	−129.40	C
		0	0	0	0.004	−177.92	0	+129.40	D

**Table 2:** Laboratory tower experiments under normal and faulty conditions.

Experiment	Condition	Cable Displacement (m)			
		1	2	3	4
0	Normal	0	0	0	0
1	Fault 1	0.004	0	0	0
2	Fault 2	0.008	0	0	0
3	Fault 3	0.012	0	0	0
4	Fault 4	0	0.004	0	0
5	Fault 5	0	0.008	0	0
6	Fault 6	0	0.012	0	0
7	Fault 7	0	0	0.004	0
8	Fault 8	0	0	0.008	0
9	Fault 9	0	0	0.012	0
10	Fault 10	0	0	0	0.004
11	Fault 11	0	0	0	0.008
12	Fault 12	0	0	0	0.012

**Table 3:** Test data confusion matrix.

		Predicted Value									
**Real** **Value**		**0**	**1**	**2**	**3**	**4**	**5**	**6**	**7**	**8**	
	**0**	10	0	0	0	0	0	0	0	0	
	**1**	0	10	0	0	0	0	0	0	0	
	**2**	0	0	10	0	0	0	0	0	0	
	**3**	0	0	0	10	0	0	0	0	0	
	**4**	0	0	0	5	5	0	0	0	0	
	**5**	0	0	0	0	0	10	0	0	0	
	**6**	0	0	0	0	0	0	10	0	0	
	**7**	0	0	0	0	0	0	0	10	0	
	**8**	0	0	0	0	0	0	0	0	10	0.94

**Table 4:** Test data confusion matrix.

Predicted Value															
**Real Value**		**0**	**1**	**2**	**3**	**4**	**5**	**6**	**7**	**8**	**9**	**10**	**11**	**12**	
	**0**	96	0	0	0	0	0	0	0	0	0	0	0	0	
	**1**	0	18	0	0	0	0	0	1	0	0	0	0	0	
	**2**	0	0	16	0	0	1	0	0	0	0	0	2	0	
	**3**	0	0	0	15	0	0	0	0	0	0	0	0	0	
	**4**	0	3	0	0	16	0	0	0	0	0	0	0	0	
	**5**	0	0	2	0	0	23	0	0	0	0	0	0	0	
	**6**	0	0	0	0	0	0	27	0	0	0	0	0	0	
	**7**	0	1	0	0	1	0	0	17	0	0	0	0	0	
	**8**	0	0	2	0	0	0	0	0	26	0	0	0	0	
	**9**	0	0	0	0	0	0	0	0	0	19	0	0	0	
	**10**	0	0	0	0	2	0	0	0	0	0	17	0	0	
	**11**	0	0	0	0	0	0	0	0	1	0	0	18	0	
	**12**	0	0	0	0	0	0	0	0	0	0	0	0	19	0.95

## Data Availability

The paper datasets are available through direct email request to the authors.

## Abbreviations

UNICAMP	Universidade Estadual de Campinas
LabEDin	Laboratório de Ensaios Dinâmicos
